# Safety and Localization of Mesenchymal Stromal Cells Derived from Human Adipose Tissue-Associated Hyaluronic Acid: A Preclinical Study

**DOI:** 10.1155/2020/1823427

**Published:** 2020-02-14

**Authors:** Janaína José dos Santos Machado, Bernard Gomes Piñeiro, Isalira Peroba Ramos, Sergio Augusto Lopes de Souza, Bianca Gutfilen, Maria Helena Nicola, Paulo Roberto Cotrim de Souza, Eduardo Cruz, Regina Coeli Goldenberg

**Affiliations:** ^1^Cryopraxis Criobiologia, Rio de Janeiro, RJ, Brazil; ^2^Departamento de Radiologia, Faculdade de Medicina, Universidade Federal do Rio de Janeiro, Rio de Janeiro, RJ, Brazil; ^3^Centro Nacional de Biologia Estrutural e Bioimagem, Universidade Federal do Rio de Janeiro, Rio de Janeiro, RJ, Brazil; ^4^Instituto de Biofísica Carlos Chagas Filho, Universidade Federal do Rio de Janeiro, Rio de Janeiro, RJ, Brazil; ^5^Hospital Geral de Bonsucesso, Rio de Janeirou7, RJ, Brazil

## Abstract

Millions of plastic surgeries are performed worldwide every year with the objective of correcting lipodystrophies stemming from lesions, tumor resections, birth defects, and AIDS-associated antiretroviral therapy. Besides that, a large number of clinical research have assessed the outcome of procedures that rely on combinations of dermal fillers and autologous cells. However, little is known about the safety of these combinations and the localization of the injected cells. The aim of this study was to test the toxicity of a solution containing 1% hyaluronic acid (HA) and adipose-derived stromal cells (ASCs) from the human adipose tissue and to assess the localization of the injected cells, with and without HA, labeled with technetium-99m. Rats received subcutaneous and intraperitoneal injections of a solution containing 1% HA/adipose-derived stromal cells isolated from the human fat tissue. The animals were then observed for up to forty-two days. The solution tested in this study did not result in systemic, biochemical, or anatomic alterations that could represent toxicity symptoms. The association of HA and ASCs labeled with technetium-99m remained at the site of the injection within a period of twenty-four hours, as demonstrated by a whole-body imaging software fusion of SPECT and CT. In conclusion, our study shows that the subcutaneous and intraperitoneal injection of HA associated with adipose-derived stromal cells (ASCs) is safe. The association of HA and ASCs did not induce local or systemic toxicity. Thus, the administration of volume equal to or less than 0.2 mL of the agent filler (1 × 10^6^ ASC+HA 1%) should be considered for subsequent studies and may be an alternative to dermal fillers due to the expected lasting effects.

## 1. Introduction

Lipodystrophy syndromes include a heterogeneous group of rare disorders clinically characterized by partial or total absence of subcutaneous adipose tissue and fat deposits in nonadipose tissues such as the liver, muscle, kidney, and pancreas. Recently, facial lipodystrophy has become a significant public health concern because of its association with the antiretroviral therapy used in the treatment of AIDS [1–6]. So far, repair surgery involves the transplant of autologous fat, dermis-fat grafts, free-flap surgery, and the use of dermal fillers [7, 8].

Autologous tissue derived from liposuction procedures and other biomaterials represent two different forms of dermal fillers [9]. Hyaluronic acid (HA) provides a biocompatible alternative for the reconstitution of connective tissue, playing a structural role in the adult skin and connective tissue [10–13].

Several papers have shown the potential benefits of human adipose-derived stem/stromal cells (ASCs) in preclinical and clinical trials; however, peer-reviewed data on ASCs have been still limited in the field of aesthetic medicine [14]. Also, hyaluronic acid, currently the filling product used in aesthetic medicine, presents significant limitations on dermal fillers, including immune reaction and longevity, and some groups have suggested that the ideal dermal filler has not yet been produced [15].

In recent years, several authors have published results related to the efficacy of using adipose-derived stem cells associated with different biomaterials in the treatment of different lesion cartilage defects [16] and osteochondral [17] and nerve regeneration [18]. According to these authors, this association would increase the regenerative efficacy.

The enrichment of autologous fat tissue and biomaterials with stem cells potentiates *in situ* generation of newly differentiated cells and the production of extracellular matrix. As a result, these combinations have longer lasting effects on patients than the use of dermal fillers alone [19–25]. Nowacki et al. demonstrated that adipose stem cell-based formulations of dermal fillers produce greater filling effects that persist significantly longer than dermal fillers prepared without ASCs. Moreover, ASCs and their soluble factor function in protective and regenerative roles in the skin, inducing collagen synthesis, inhibiting melanogenesis, and recruiting and protecting dermal fibroblasts [15].

Several clinical studies have focused on the use of autologous-derived stem cells in plastic surgery. However, little is known about the localization of injected stem cells and the potential side effects of their use [26].

Adipose-derived stem cells proliferate rapidly with a few passages and exhibit a stable phenotype after the third passage. These properties allowed us to obtain a large number of ASCs with a low risk of culture-induced chromosomal abnormalities or teratoma formation because the latter typically is not associated with mesenchymal stem cells [19, 20]. According to Lequeux et al., the advantages of using ASCs rather than adipose tissue are numerous. This technique is reproducible and controllable, since an exact number of cells can be injected and phenotype is well known. Indeed, the cell suspension contains more than 95% of ASCs expressing the mesenchymal markers CD105, CD90, and CD73 and less than 5% cells express the hematopoietic-related markers, CD14 and CD45. This newly formed adipose tissue, when used in cosmetic surgery, could restore skin volume and therefore attenuate or even lead to a more durable disappearance of wrinkles than that obtained by injecting HA, which only has a transient effect since it is resorbed over time [27].

Based on the data that support the additional benefits demonstrated by the association of HA and ASCs, the present study used animal models to test the toxicity of a solution containing 1% hyaluronic acid (HA) and adipose derived stromal cells (ASCs). Furthermore, using the same model, we assessed the localization of the injected ASCs with HA labeled with the radioisotope technetium-99m (^99m^Tc) to determine if HA interferes with its localization and performance. ^99m^Tc-ASCs were assessed by whole-body imaging software fusion of SPECT and CT at about twenty-four hours after the injection.

The rationale of the study was to evaluate the safety and localization of cells present in a new advanced cellular therapy product. Cell-based products have been used indiscriminately for decades, but only a few years ago, the world's regulatory agencies began to issue regulations for the manufacture of this type of product. Due to the plasticity of ASCs, it is essential that the nonclinical and clinical studies are performed with well-defined and characterized processes. These products intended for clinical use should be produced via a robust manufacturing process governed by quality control enough to ensure consistent and reproducibility [28, 29]. One of the main goals of regenerative and personalized medicine is the development of cellular therapies free of side effects and devoid of ethical concerns.

## 2. Materials and Methods

### 2.1. ASC Obtention and Characterization

The adipose tissues were obtained from healthy patients (twenty women, aged between thirty and sixty) submitted to aesthetic liposuction procedures at the outpatient clinic for dermatological surgery of the Dermatology Service of the Bonsucesso Federal Hospital (HFB), Rio de Janeiro, Brazil. All volunteers signed an informed consent form, according to the procedures approved by the local ethics committee under protocol number 30/10 of the CEP/CONEP platform.

The ASCs were isolated and cultured as described previously in the literature [19, 25]. Briefly, fat tissue was subjected to enzymatic digestion with Collagenase Type II 0.01% (Worthington-Biochem, Lakewood, NJ, USA) at 37°C, under agitation. Then, the stromal vascular fractions (SVF) isolated were plated at 1.0 × 10^7^ cells per 75 cm^2^ bottle (TPP, Trasadingen, Switzerland) in supplemented DMEM-LG medium (LGC, São Paulo, SP, Brazil). After reaching 80% of confluence, the ASCs were enzymatically detached, and these ASCs were used in the third passage.

In order to confirm if the obtained cells after the culture were indeed ASCs, the cells were labeled with antibodies against specific surface antigen. Surface antigen expression allows a rapid identification of cell population. Immunophenotypes were determined through the evaluation of specific surface antigen expression as described by Dominici et al. [30]: hematopoietic cells (CD45—BD Pharmigen, San Jose, CA, USA), mesenchymal cells (CD105, CD73, and CD90—BD Pharmigen, San Jose, CA, USA), and endothelial cells (CD31, CD133—BD Pharmigen, San Jose, CA, USA). The labeled cells were acquired in a BD FACS ARIA IIu-flow cytometer (Becton Dickinson, San Diego, CA, USA), and at least fifty thousand events were collected and analyzed. The cellular plasticity was assessed by the induction of cell differentiation into osteocyte, adipocyte, and chondrocyte lineages following the methodology described by Zuk et al. [19]. To state it briefly, the ASCs were plated on microscopy coverslips in 35 × 10 mm Petri dishes (Corning, Steuben, NY, USA) at a density of 3 × 10^2^ cells/cm^2^. When the cultures reached a confluence of 70%, a differentiation induction medium [19] was substituted for the culture medium.

### 2.2. ASC Seeding in HA: Evaluation of Cell Adhesion and Morphology

To test *cell adhesion and morphology*, 1 × 10^6^ ASCs were diluted at a rate of 1 : 1 with 2% HA (Silvestre Labs. Química e Farmacêutica Ltda., Rio de Janeiro, RJ, Brazil). This cell suspension was kept in culture and analyzed in three different times after plating (one hour, twenty-four hours, and seven days). As a negative control, a cell suspension of 5 × 10^5^ cells/mL was kept in culture under the same conditions in DMEM-LG supplemented with 10% FCS.

### 2.3. ASC Association with the Biomaterial

To prepare the filler agent,2 × 10^6^ cells were suspended by a reciprocal mix in 1 mL of experimental solution containing 2% of hyaluronic acid (Silvestre Labs Química e Farmacêutica Ltda., Rio de Janeiro, Brazil) diluted in NaCl 0.9% (Equilex, Rio de Janeiro, RJ, Brazil), reaching a final cell of concentration 1.0 × 10^6^ cells and 1% of HA.

### 2.4. Time-Course Analysis of Hypersensitivity and Systemic Toxicity

The safety of the filler agent (HA/ASC) was analyzed through tests of hypersensitivity, irritation, and toxicity, performed based on ISO 10993 [31, 32], in accordance with the recommendations of consensus on the classification of adverse events related to HA at the time of initiation.

As test systems, one hundred and fifty (150) *Rattus norvegicus*, Wistar lineage, males, between twelve and fourteen weeks of age, were used at the beginning of the administration.

The evaluation of hypersensitivity and systemic toxicity of the filler agent (HA/ASC), as well as all its components, in an individualized way (NaCl 0.9%, hyaluronic acid 1%, adipose-derived stem cell), was carried out, having Freund's adjuvant complete (Sigma-Aldrich, St. Louis, MO, USA) as positive control. The test system was prepared for administration by shaving and disinfecting the area. The test substances were administered once in the subcutaneous tissue of the cervicodorsal region, in a fixed volume of 0.2 mL/test system. In parallel, a positive control group was added to the assay, receiving a fixed volume of 0.2 mL (0.05 mL of FAC in 0.15 mL saline) per test system.

The same test substances and administration type were evaluated for hypersensitization and systemic toxicity in the rats (*n* = 75 per assay). In the first assessment, the animals were closely observed for the first three days for clinical parameters such as alterations in the hair, skin, eyes, mucosa, respiration, motor activity, behavior, and for the occurrence of adverse effects such as tremors, convulsions, salivation, diarrhea, lethargy, sleepiness, and coma.

At the end of the first assay, the animals were euthanized through deep sedation and examined macroscopically for the evaluation of possible toxic effects. Histological evaluations of the injection site were performed for the evaluation of inflammatory reactions.

Afterwards, the same number of animals (*n* = 75) was submitted to similar procedures and was assessed on the fourteenth, twenty-eighth, and forty-second days after the injection for analysis of systemic toxicity. At the end of each observation period, the animals fasted overnight prior to blood collection from the hepatic vein, and then euthanized through profound sedation. Hematology and biochemical profile blood tests were performed.

### 2.5. Assessment of Cellular Localization with Technetium-99m

Briefly, 1 × 10^7^ cells were incubated for ten minutes at room temperature in 500 *μ*L of stannous chloride (SnCl_2_). Subsequently, 25 mCi of ^99m^Tc were added to the solution, and the incubation continued for an additional ten minutes. The mixture was then centrifuged at 500xg for five minutes, and the pellet was suspended in NaCl 0.9% solution. The viability of the labeled cells was assessed by the Trypan blue (Sigma-Aldrich, St. Louis, MO, USA) exclusion test. Labeling efficiency (percentage) was calculated by the activity in the pellet divided by the sum of the radioactivity in the pellet plus supernatant. To determine if HA interferes in the localization of the cells, one group of mice (*n* = 5) received an injection, subcutaneously and in the dorsal region, of 0.2 mL of ^99m^Tc-ASC (1 × 10^6^ cells) associated with 1% HA in saline solution; another group (*n* = 5) received an injection of 0.2 mL ^99m^Tc-ASC (1 × 10^6^ cells) without HA.

Whole-body, planar, and SPECT scintigraphy was carried out three hours and twenty-four hours after cell infusion in a GE gamma-camera (GE Healthcare, Chicago, IL, USA) and a computed tomography was performed in an Optima PET/CT 560 (GE Healthcare, Chicago, IL, USA). A SPECT/CT fusion was processed with the OsiriX© Software.

### 2.6. Statistical Analysis

The data were analyzed with the ANOVA analysis of variance, followed by the *post hoc* Bonferroni procedure. Differences were considered significant when *p* < 0.05.

## 3. Results

### 3.1. ASC Obtention from Adipose Tissue

**Figure 1:**
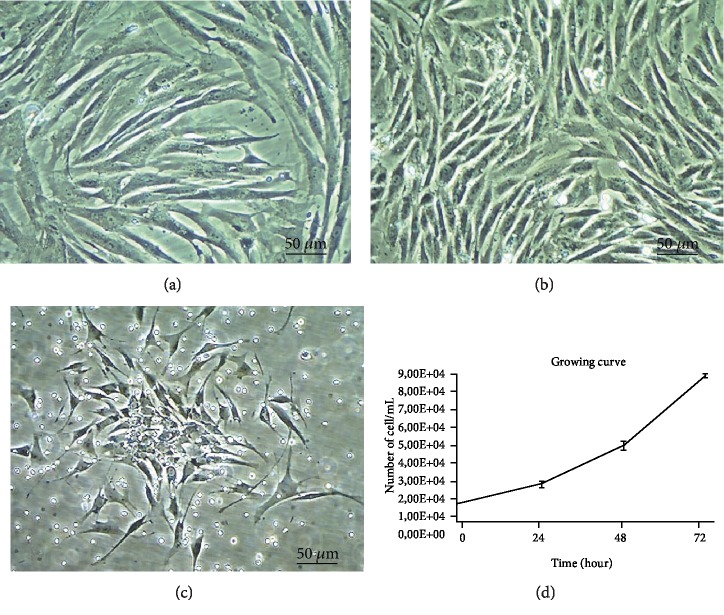
Characterization of ASC in the third passage. (a) Morphology of cultured ASC three days of culturing and (b) ASCs seven days of culturing. All ASCs exhibited spindle-shaped morphology. Scale bar = 50 *μ*m (magnification: 100x). (c) ASCs were assessed for clonogenic ability by colony-forming unit-fibroblast (CFU-F) and (d) growth curve.

The ASCs obtained in this study presented high capacity of adhesion to the plastic of the culture flasks, high proliferation potential, and exhibited a fibroblastic-like aspect in the third passage (Figures 1(a)–1(c)).The cells showed exponential growth, by doubling their population approximately every twenty-four hours (Figure 1(d)).

### 3.2. Cellular Differentiation into Mesodermal Lineages

**Figure 2:**
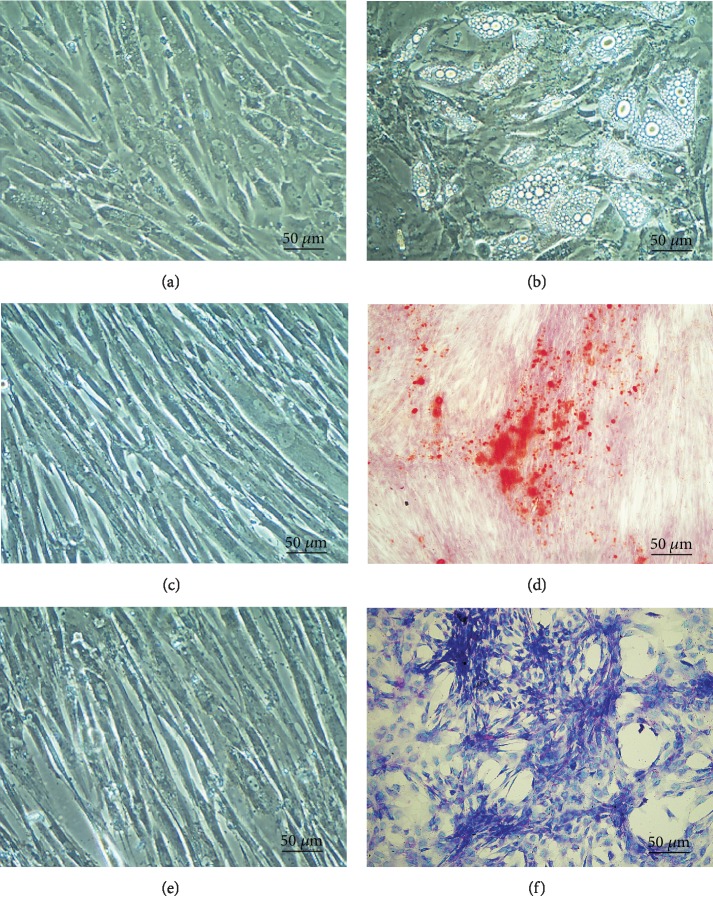
Analysis of the differentiation capacity of ASC. (a, b) Photomicrography on inverted microscope of phase contrast (magnification: 100x). (a) Negative control ASC in DMEM media and (b) differentiated ASCs presenting lipid vacuoles (magnification: 100x). (c, d) Cells stained with 1% Alizarin red, shown in (c) negative control and (d) differentiated ASCs presenting calcium deposits (magnification: 40x). (e, f) Cells stained with Alcian blue, in (e) negative control and (f) ASCs differentiated presenting the accumulation of sulfated proteoglycans (magnification: 100x).

The ASCs were differentiated into adipogenic, osteogenic, and chondrogenic lineages. When cultivated in an adipogenic induction medium, these cells started to present many vacuoles that represent the accumulation of lipid characteristic of the multilocular adipose tissue (Figure 2(b)) when compared to the control group (Figure 2(a)).

In order to confirm osteogenic differentiation, calcification of extracellular matrix was analyzed in the ASC cells by the use of Alizarin red. When compared to the control group (Figure 2(c)), calcification can be seen as red regions within the cell monolayer (Figure 2(d)). Also, in order to confirm chondrogenic differentiation, the cells were cultivated with chondrogenic induction media for twenty-one days. After this period, the cells presented an accumulation of sulfated proteoglycans which could be specifically detected by using the stain Alcian blue under acidic conditions (Figure 2(f)), compared to the control group cultured with the standard medium (Figure 2(e)).

### 3.3. Immunophenotyping

**Figure 3:**
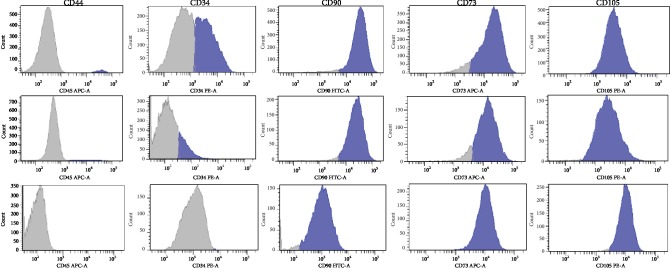
Modification of expression of specific surface antigen along passages in adipose mesenchymal stromal cells. (a) First passage. (b) Second passage. (c) Third passage. It is possible to visualize the presence of hematopoietic cells (CD45^+^) and precursor cells (CD34^+^) at the beginning of the culture. Throughout the passages, the population of mesenchymal cells became more homogeneous; besides that, they express similar levels of CD73 and CD105 specific surface antigens, with reduction of the coefficient of variation (width) of histograms.

After the third passage, the ASCs presented cluster differentiation expression: CD45^−^CD34^−^, CD105^+^, CD73^+^, and CD90^+^ (Figure 3(c)). The adherent cells expressed more than 95% of the human mesenchymal stromal cell markers CD105, CD90, and CD73. These cells also presented low expressiveness for the hematopoietic markers CD34 and CD45. Figures 3(a) and 3(b) show the presence of hematopoietic cells in the first and second passages.

### 3.4. Cellular Adhesion and Morphology

**Figure 4:**
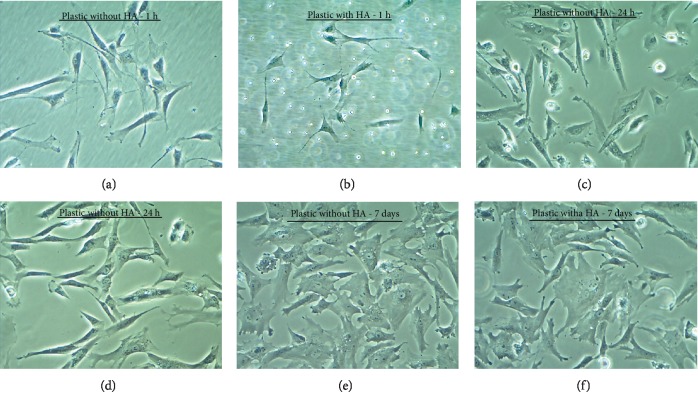
ASC seeding HA evaluation of cell adhesion and morphology. (a) Adhesion of the cells to plastic in the medium not supplemented with the biomaterial (HA) one hour after culture. (b) Cell adhesion in plastic in the medium containing 1% HA one hour after culture. (c) Adhesion of the cells to plastic in the medium not supplemented with the biomaterial (HA) twenty-four hours after culture. (d) Cell adhesion in plastic in the medium containing 1% HA twenty-four hours after culture. (e) Adhesion of the cells to plastic in the medium not supplemented with the biomaterial seven days after culture. (F) Cell adhesion in plastic in the medium containing 1% HA seven days after culture. Photomicrography on an inverted microscope of phase contrast (magnification: 100x).

There was no *in vitro* evidence of cellular adhesion and morphology alterations among the evaluated groups ASC+HA (Silvestre Labs, RJ, Brazil), when compared with the control group ASC+DMEM-LG. The cultures presented similar characteristics regarding cellular adhesiveness and morphological aspect. These parameters were analyzed one hour, twenty-four hours, and seven days after culture (Figure 4).

### 3.5. Assessment of Hypersensitivity

**Figure 5:**
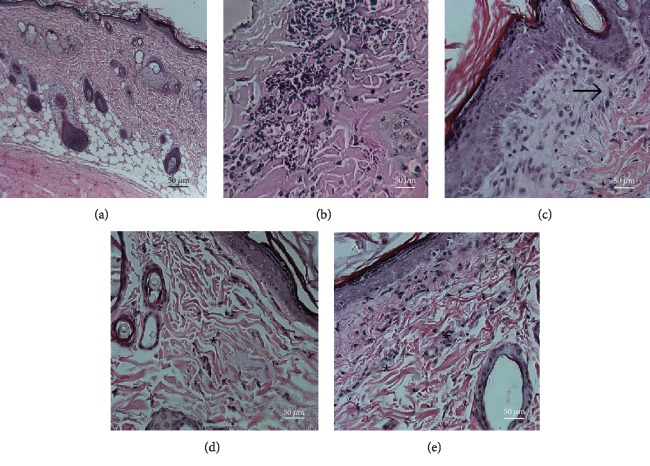
Microscopic analysis of subcutaneous tissue after injection of test substances: (a) Negative control; infusion of 0.9% NaCl; (b) positive control–FAC; (c) HA1% in0.9% NaCl; (d) ASC; (e) ASC+HA (hematoxylin-eosin, 40x).

There were no statistically significant alterations in the clinical parameters evaluated, such as skin and hair disorders, alteration of eyes and mucous membranes, respiratory and circulatory changes, central nervous system disorders, changes in somatomotor activity and standard behavior, tremors, convulsions, salivation, diarrhea, lethargy, drowsiness, and coma. Macroscopic and microscopic analysis showed inflammation to varying degrees in animals treated with either HA, complete Freund's adjuvant, or filler agent (ASC+HA), as expected (Figure 5 and Table 1).

### 3.6. Assessment of Systemic Toxicity

There were no statistically significant alterations in macroscopic parameters, body weight gain (Table 2), as well as water (Table 3) and feed consumption (Table 4) among the filler agent group or any of the four control groups during the observation days. Hematology and biochemical profile blood tests showed no signs of acute toxicity (Tables 5 and 6), and the subcutaneous injection of the tested substances did not cause deaths in the evaluated animals.

### 3.7. Assessment of Cellular Localization with Technetium-99m

**Figure 6:**
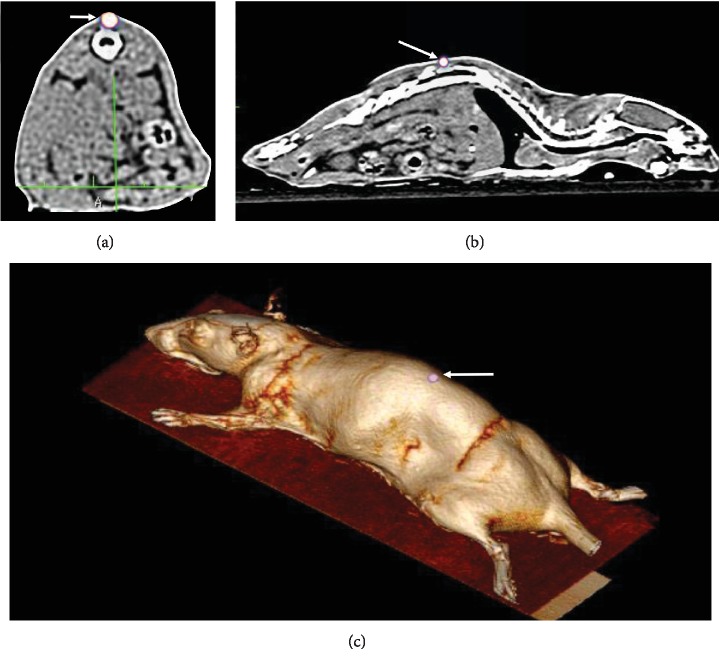
SPECT and CT of ASC labeled with ^99m^Tc. Representative whole-body imaging software fusion of SPECT and CT acquired twenty-four hours after subcutaneous administration of ASC labeled with ^99m^Tc and associated with 1% HA. White arrows point to ^99m^Tc-ASC. (a) Transaxial slice. (b) Sagittal slice. (c) 3-D reconstruction SPECT/CT images. Arrow = ^99m^Tc-ASC.

The labeling efficiency of ^99m^Tc-ASCs was 85% and labeled cell viability was up to 95%. The labeling procedure did not affect viability of cells. As shown in Figure 6, labeled ^99m^Tc-ASCs (1.0 × 10^6^ cells) associated with 1% HA cells remained at the site of injection for a period of twenty-four hours and no signs of migration to any organs were detected.

## 4. Discussion

The success of plastic surgeries involving a combination of substrate and stem cells depends on the biocompatibility of the substrate and on whether cells remain or not at the site of injection. It has been supported that the use of a combination of mesenchymal stem/stromal cells with an adequate 3D matrix enhances their regenerative efficacy [16]. Also, it is shown that the microenvironment stiffness and elasticity are important for stem cell differentiation and the form of tissue shaped by the cells [33].

The enrichment of autologous fat tissue and biomaterials with stem cells potentiates *in situ* generation of newly differentiated cells, and the production of extracellular matrix (ECM). As a result, these combinations have longer lasting effects on patients than dermal fillers alone [19–25]. Adipose-derived stem cells and fat grafts have been used in clinical research of hemifacial and/or lipoatrophy [7, 34–36] and other aesthetic treatments [8, 13, 37–39].

Some other clinical studies showed the potential use of a combined treatment composed of platelet-rich plasma (PRP) and hyaluronic acid (HA) to stimulate the regeneration of wounds [40–42]. Also, in a burn wound model in rat, a combination of hyaluronic acid and adipose-derived stem cells (ASCs) was able to stimulate wound healing [43].

In consonance to *in vivo* model, ASCs cultured in a 3D HA gel show the potential of the spheroids for promoting tissue regeneration [44]. In addition, ASCs cultured in a 3D collagen type I scaffold combined to PRP and human recombinant insulin might be useful to treat osteochondral defects [45]. As described, the ASCs can be a very useful tool in tissue repair, because these cells produce several cytokines and anti-inflammatory, immunomodulatory, antiapoptotic, and angiogenic substances, as well as neurotrophic factors [46].

Hyaluronic acid is a resorbable filler widely used in the cosmetics industry, especially in cosmetic surgery giving support, volume, and moisture to the tissue [47–49]. The aesthetic effects of this product are considered transitory, for its natural and progressive degradability depends on native hyaluronidase, and it is expected that its reabsorption occur over a 12- to 18-month period [50]. Hypersensitivity reactions are rare, but when they occur, they may cause angioedema after the injection [51, 52].

Although more extensive studies must be done in the aesthetic medicine using dermal filler, this field is growing significantly and new cell-based product need to be analyzed before its clearance [28, 29, 53]. One of the main goals of regenerative and personalized medicine is the development of cellular therapies free of side effects and devoid of ethical concerns [54]. Stem cell-based products intended for clinical use should be produced via a robust manufacturing process governed by quality control enough to ensure consistent and reproducible final product. Nonclinical evaluation for stem cell-based medicine should be done in order to adequately evaluate different aspects including proof of concept, localization, immune rejection, and safety [28, 29, 53]. In this scenario, our study investigated the safety and the localization of ASCs associated with experimental HA produced in Brazil. In this research, we used a healthy rat model to test the toxicity of a filler agent, solution containing 1% HA, and ASCs derived from the human adipose tissue. In addition, we evaluated the localization of HA/ASCs solution labeled with ^99m^Tc.

Our results indicate that subcutaneous administration of the test solution, HA, ASCs, and FAC did not cause mortality or changes in weight gain during the observation period. Animals in the HA/ASCs and HA displayed an increased volume at the site of injection. As previously described [55], animals treated with Freund's adjuvant complete (FAC) displayed signs of inflammation and had bristly hair, erythema, and swelling at the site of injection. The biochemical and hematological parameters assessed remained within described reference values [56, 57]. Macroscopic analyses did not reveal significant alterations among groups, which can be considered normal. Mild erythema appeared in animals which received HA, disappearing within three days after application [49]. Also, moderate swelling is usually found in cosmetic applications [48]. Skin damage involves a number of different degenerative processes, notably a decrease in collagen production by fibroblasts. Several cytokines and growth factors are involved in stimulating fibroblast collagen synthesis for skin regeneration and have been shown to be a part of the molecules secreted by ASCs, suggesting that these cells may be suitable for promoting repair of atrophic and damaged skin [7]. The likely mechanism of action includes paracrine activation of dermal fibroblasts and dermal angiogenesis. Thus, the maintenance of the cells at the injection site is optimal to support regeneration of tissue due to the ability of the cells to secrete factors that promote the regeneration of the adjacent tissue [58].

Regarding the localization of stem cell, the use of nuclear medicine in the evaluation of cell displacement has been widely used in various types of studies to track the migration of cells; many imaging methods such as PET, SPECT, and MRI have been used [59–61]. In our studies, we used ^99m^Tc-ASCs to evaluate cell homing. The choice of the ^99m^Tc was due to its characteristics such as short decay time, easy image capture through the use of gamma cameras ensuring optimum image quality, and high sensitivity and availability when compared to other types [62–65]. Our results showed that ^99m^Tc-ASCs+HA remained at the injection site for a period of twenty-four hours (Figure 6), with no signs of dispersion or migration to different organs, showing its feasibility. Feng et al. had demonstrated that labeling signals of allogeneic ASCs could be detected by MRI 14 weeks after intra-articular injection into the osteoarthritis sheep [66]. Although our labeling technique allowed us to image the labeled cells only up until twenty-four hours after injection, we clearly observed that the cells remained at the site of injection.

Several authors have demonstrated in their published articles the permanence of cells associated with biomaterials at the injection site. The retention of these cells at the injection site is minimized by adhesion of these cells to biomaterials [15, 66–69]. According to Bertozzi et al., the use of HA could prevent excessive ASC diffusion, allowing sustained release at the injection site [70]. Smith et al. [71], in an *in vivo* study using a mouse model of MI, observed that an injection of HA-based material approved for cell therapy that is used in combination with stem cells dramatically increases cell retention 24 h after delivery when compared with cells in PBS. Even in long-term cell engraftment (3 weeks after delivery), a significant retention is observed. The survival effect observed after 3 week post-transplantation is attributed to cell material interactions through the CD44 receptor.

The number of experimental studies evaluating the use of cells associated with biomaterials as dermal fillers is still small; few researchers have evaluated cell migration or described the practical and clinical applications of this type of cell therapy. The *in vivo* effect of cell-based dermal fillers cannot be considered fully safe until the factors that lead to local cell persistence are more thoroughly evaluated and understood [72–74].

Further preclinical studies with standardized protocols and larger randomized clinical trials according to international guidelines, such as those stipulated by the FDA, are still needed to ensure safety and efficacy use of any cell-based products before these cells can be used clinically [75]. In particular, researchers need to demonstrate the safety and effectiveness of ASCs in animal models, alone or in combination with new biomaterials [26]. Therefore, before clinical use, many issues such as in situ stability, differentiation, and cell migration should be addressed [76].

## 5. Limitation

The limitations of our model include follow-up periods of twenty-four hours, which may be insufficient to demonstrate the long-term permanence of the cells in the site of injection and consequently its influence surrounding host tissue. The injection of a filler agent in lipodystrophy rat models would allow us to analyze its efficacy.

## 6. Conclusion

In summary, the present work was designed as a preclinical study to assess the safety and localization of a dermal filler agent containing human ASCs+HA in animal models. Despite the reported changes, the association of a biomaterial and human cells tested here did not result in systemic, biochemical, or anatomic alterations that could represent toxicity symptoms. In our study model, it has been shown that the agent filler (ASCs+HA) is safe in subcutaneous and intraperitoneal injections.

Moreover, cells remained at the site of injection as required for the restoration and enhancement of form, as well as the continued long-term maintenance of aesthetic results. Thus, the administration of volumes equal to or less than 0.2 mL of the agent filler (1 × 10^6^ ASCs+HA 1%) should be considered for subsequent studies. To the best of our knowledge, it is the first time that this kind of study has been carried out and described in the literature.

## Figures and Tables

**Table 1:** Summary of the evaluation of the macroscopic alterations presented by the test systems after subcutaneous injection of the test substances.

Observation	Time	30 min.					24 hours					48 hours				
	Animals/group	Saline	HA 1%	ASC	ASC+HA1%	FAD	Saline	HA 1%	ASC	ASC+ HA1%	FAD	Saline	HA 1%	ASC	ASC+HA1%	FAD
Bristly hair	5	0%	0%	0%	0%	100%	0%	0%	0%	0%	100%	0%	0%	0%	0%	100%
Discrete subcutaneous edema	5	20%	20%	20%	20%	80%	0%	0%	0%	20%	100%	0%	0%	0%	20%	100%
Subcutaneous increase in volume	5	0%	100%	0%	100%	80%	0%	100%	0%	100%	80%	0%	100%	0%	100%	80%
Subcutaneous petechiae	5	0%	0%	20%	20%	80%	0%	0%	0%	0%	60%	0%	0%	0%	0%	0%
Subacute inflammatory infiltrate in the deep dermis	5	0%	0%	20%	0%	100%	0%	0%	0%	0%	100%	0%	0%	0%	20%	100%
Discrete subacute dermatitis in the deep dermis	5	0%	0%	0%	20%	100%	0%	0%	0%	20%	80%	0%	0%	0%	20%	80%
Discrete subacute dermatitis in the superficial dermis	5	0%	0%	0%	0%	100%	0%	0%	0%	0%	80%	0%	0%	0%	0%	20%
Severe subacute dermatitis in the deep dermis	5	0%	0%	0%	0%	20%	0%	0%	0%	0%	20%	0%	0%	0%	0%	20%
Discrete chronic dermatitis in the deep dermis	5	0%	0%	0%	0%	0%	0%	0%	20%	0%	80%	0%	0%	0%	0%	0%
Moderate chronic dermatitis in the deep dermis	5	0%	0%	0%	0%	0%	0%	0%	20%	0%	80%	0%	0%	0%	0%	0%

**Table 2:** Assessment of body-weight change in test systems followed up for six weeks after subcutaneous infusion of test substances.

Weeks	Body weight (g)										
	Groups	Saline (NaCl)		ACS		HA 1%		ASC + HA 1%		FAC	
	Animals (*n*)	Media	SD	Media	SD	Media	SD	Media	SD	Media	SD
1	5	295	31	341	63	340	27	343	27	362	25
2	5	328	39	354	43	348	22	360	30	370	31
3	5	353	42	370	38	351	24	375	26	380	31
4	5	366	39	384	27	360	23	385	28	392	35
5	5	381	44	399	22	374	22	387	22	391	33
6	5	390	47	407	16	380	21	404	25	407	38

*N*: no. of animals; NaCl: sodium chloride; HA: hyaluronic acid; ASC: adipose-derived stromal cells; FAC: Freund's adjuvant complete. ASC+HA 1%. ANOVA with Bonferroni's correction (Bonferroni's post hoc test).

**Table 3:** Evaluation of the change in the water consumption of the test systems assessed for up until six weeks after the subcutaneous infusion of the test substances.

Weeks	Water consumption (mL)										
	Groups	Saline (NaCl)		ACS		HA 1%		ASC+HA 1%		FAC	
	Animals (*n*)	Media	SD	Media	SD	Media	SD	Media	SD	Media	SD
1-2	5	250,80	25,74	255,60	2036	286,20	3,11	236,60	34,22	234,40	22,63
1-4	5	218,25	68,31	241,25	14,30	253,40	15,83	259,00	35,49	248,50	39,94
1-6	5	281,17	59,34	269,37	30,93	242,40	34,04	226,93	39,67	319,00^∗^	65,70

*N*: no. of animals; NaCl: sodium chloride; HA: hyaluronic acid; ASC: adipose derived stromal cells; FAC: Freund's adjuvant complete. ASC+HA 1%. ANOVA with Bonferroni's correction (Bonferroni post hoc test). ∗*p* < 0, 05 FAC vs. ASC+HA 1%.

**Table 4:** Evaluation of changes in feed consumption of the accompanying test systems for up to six weeks after subcutaneous infusion of test substances.

Weeks	Feed consumption (g)										
	Groups	Saline (NaCl)		ACS		HA 1%		ASC+HA 1%		FAC	
	Animals (*n*)	Media	SD	Media	SD	Media	SD	Media	SD	Media	SD
1-2	5	155,00	12,73	172,00	24,04	168,00	21,21	190,90	1,27	157,40	17,54
1-4	5	155,55	10,37	166,05	10,88	159,30	13,27	164,60	17,26	161,00	26,72
1-6	5	174,37	22,03	189,60	23,17	161,27	15,88	167,67	25,14	159,80	25,31

*N*: no. of animals; NaCl: sodium chloride; HA: hyaluronic acid; ASC: adipose-derived stromal cells; FAC: Freund's adjuvant complete. ASC+HA 1%. ANOVA with Bonferroni's correction (Bonferroni's post hoc test).

**Table 5:** Results of the complete blood count of the test systems after subcutaneous injection evaluated on the fourteenth, twenty-eighth, and forty-second days.

Blood count	Animals	14 days					28 days					42 days				
		Saline	HA 1%	ASC	ASC+HA 1%	FAC	Saline	HA 1%	ASC	ASC+HA 1%	FAC	Saline	HA 1%	ASC	ASC+HA1%	FAC
	n	Media	Media	Media	Media	Media	Media	Media	Media	Media	Media	Media	Media	Media	Media	Media
RBC (mm^3^)	5	8,12	7,86	8,05	8,7	8,14	10,88	10,99	10,36	10,7	11,36	10,88	10,99	10,36	10,7	11,36
HTC (g/dL)	5	53,6	54,5	54	57	55,4	59,2	59,4	57	59	61,8	59,2	59,4	57	59	61,8
Hb (g/dL)	5	17,4	18,1	17,8	19,62	18,84	20,96	20,92	19,82	20,62	21,82	20,96	20,92	19,82	20,62	21,82
MCV (fL)	5	66,01	69,42	67,08	65,6	68,1	54,47	54,05	55,04	55,15	54,44	54,47	54,05	55,04	55,15	54,44
MHC (Pg)	5	21,42	23,01	22,1	22,56	23,13	19,3	19,04	19,14	19,29	19,21	19,3	19,04	19,14	19,29	19,21
MCHC (mg/dL)	5	32,45	33,14	32,93	34,39	33,97	35,43	35,22	34,77	34,96	35,29	35,43	35,22	34,77	34,96	35,29
WBC (mm^3^)	5	9.600,00	100	8480	6820	7560	8.160,00	7960	7260	8060	9500	8.160,00	7960	7260	8060	9500
Basophils (mm^3^)	5	38,8	0	0	0	0	0	0	0	0	0	0	0	0	0	0
Neutrophils (mm^3^)	5	1777,2	999,5	1505,4	1227,2	1613,6	1.382,80	1593,4	1.173,60	1203,8	1833	1.382,80	1593,4	1.173,60	1203,8	1833
Eosinophils (mm^3^)	5	69,4	58	58,2	90	85	52,2	29	12,2	18,4	52	52,2	29	12,2	18,4	52
Lymphocytes (mm^3^)	5	6578	6602,5	5698,2	4614,8	5011,2	5799	6033,6	5838,8	6252,2	7072,4	5799	6033,6	5838,8	6252,2	7072,4
Monocytes (mm^3^)	5	1157	440	1218,2	888	850,2	926	340	235,4	585,6	542,6	926	340	235,4	585,6	542,6
PCT (mm^3^)	5	710.400	785.750	758.200	802.200	703.400	1.176.200	1.287.800	1.044.400	1.109.600	1.281.200	1.176.200	1.287.800	1.044.400	1.109.600	1.281.200
TP (Seg)	5	21,9	24,02	21,2	21,96	22,54	23,24	22	22,84	23,58	22,08	23,24	22	22,84	23,58	22,08
TTPa (Seg)	5	19,1	22,42	21,74	18,4	20,3	20,9	18,96	18,16	18,58	17,06	20,9	18,96	18,16	18,58	17,06

**Table 6:** Results of biochemical tests of the test systems after injection of the test substances evaluated on the fourteenth, twenty-eighth, and forty-second days.

Biochemical analysis		14 days										28 days										42 days									
		Saline		ASC		HA1%		ASC+HA 1%		FAC		Saline		ASC		HA1%		ASC+HA 1%		FAC		Saline		ASC		HA1%		ASC + HA 1%		FAC	
	*N*	Media	SD	Media	SD	Media	SD	Media	SD	Media	SD	Media	SD	Media	SD	Media	SD	Media	SD	Media	SD	Media	SD	Media	SD	Media	SD	Media	SD	Media	SD
Albumin (g/dL)	5	3,86	0,28	3,86	0,2	3,84	0,27	4,03	0,11	3,82	0,1	4,12	0,19	4,09	0,11	4,09	0,28	4,14	0,3	4,03	0,58	4,1	0,15	4,16	0,16	4,17	0,26	4,22	0,15	4,09	0,11
ALT (UI/L)	5	55,5	26	45,3	11	39,6	8,04	45,32	9	43,76	6,51	54,56	10.69	53,8	3.84	52,2	9,99	52,62	13,1	44	11,1	49,4	9.84	56,66	13,7	64,88	25,3	55,8	6,45	37,74	2,42
AST (mg/dL)	5	106	28,2	77,44	8,45	96	9,52	100,7	6,53	77,80^∗^	12,2	77,04	8,98	71,7	10,5	67,2	12	63,2	23,1	92,54^∗^	4,21	84,7	7,91	78,2	6,39	76,32	18	82,4	7,12	85,42	10,5
Chloride (mEq/L)	5	101	0,71	100,8	0,45	103	1,48	100,2	1,48	102	1,58	100,2	1,1	101	0,55	101.60	1,14	100,6	1,14	60,2	1,1	103.00	1,58	100,6	0,55	102	1	102	1,3	102,2	1,64
Creatinine (mg/dL)	5	0,44	0,05	0,36	0,05	0,4	0,07	0,38	0,04	0,4	0	0,42	0,04	0,34	0,05	0,42	0,08	0,38	0,04	0,4	0	0.44	0,05	0,48	0,13	0,42	0,04	0,38	0,08	0,4	0,07
Total protein (mEq/L)	5	5,7	1,81	6,02	0,9	4,62	0,81	6,9	0,75	6,14	1,7	7,68	1,15	7,98	8,86	8,46	0,66	7,94	0,68	8,06	1,1	6,24	1,4	6,82	1,59	7,34	1,73	7,4	1,69	6,06	1,52
Ptn Totais (g/dL)	5	5,82	0,19	5,86	0,3	5,62	0,33	6,06	0,15	5,8	0,26	6,12	0,15	5,98	0,13	6,1	0,07	6,32^∗^	0,16	6,26	0,27	5,92	0,3	5,98	0,18	5,84	0,24	5,96	0,25	5,94	0,11
Sodium (mEq/L)	5	135	2,07	133,4	1,52	137	1,22	136,2	0,84	136,6	1,82	135	1,22	135	1,14	134,2	1,48	134,8	1,92	133	3,03	135	0,55	135,6	1,67	135,8	1,48	136	1,92	135,4	11,5
Urea (mg/dL)	5	43,1	3,02	41,65	3,65	46,1	7,29	45,71	6,71	44,33	4,59	43,32	3,83	47	6,11	42,35	4,39	46,35	6,68	43,3	6,85	58,3	12,6	45,62	5,91	48,68	6,86	43,9	2,93	46,64	4,74
Glucose (mg/dL)	5	81,2	13,7	99,6	21,3	73,8	14,3	83,4	4,98	76	2,24	98,4	11,4	83,2	8,58	88,8	7,26	77,6	9,45	82,6	17,4	88,8	7,33	94,2	23,6	83	10,7	89,8	2,95	90,2	13,2

*N*: no. of animals; NaCl: sodium chloride; HA: hyaluronic acid; ASC: adipose-derived stromal cells; FAC: Freund's adjuvant complete. ASC+HA 1%. ^∗^*p* < 0, 05*FAC vs. saline*.

## Data Availability

Authors will provide the raw data in connection with the paper for editorial review and/or for public access. The data will be retained for a reasonable time after publication.
